# An opponent model for agent-based shared decision-making via a genetic algorithm

**DOI:** 10.3389/fpsyg.2023.1124734

**Published:** 2023-10-03

**Authors:** Kai-Biao Lin, Ying Wei, Yong Liu, Fei-Ping Hong, Yi-Min Yang, Ping Lu

**Affiliations:** ^1^School of Computer and Information Engineering, Xiamen University of Technology, Xiamen, China; ^2^School of Data Science and Intelligent Engineering, Xiamen Institute of Technology, Xiamen, China; ^3^Department of Neonates, Xiamen Humanity Hospital, Xiamen, China; ^4^Department of Pediatrics, Xiamen Hospital of Traditional Chinese Medicine, Xiamen, China; ^5^School of Economics and Management, Xiamen University of Technology, Xiamen, China

**Keywords:** shared decision-making (SDM), agent, auto-negotiation, genetic algorithm, opponent model

## Abstract

**Introduction:**

Shared decision-making (SDM) has received a great deal of attention as an effective way to achieve patient-centered medical care. SDM aims to bring doctors and patients together to develop treatment plans through negotiation. However, time pressure and subjective factors such as medical illiteracy and inadequate communication skills prevent doctors and patients from accurately expressing and obtaining their opponent's preferences. This problem leads to SDM being in an incomplete information environment, which significantly reduces the efficiency of the negotiation and even leads to failure.

**Methods:**

In this study, we integrated a negotiation strategy that predicts opponent preference using a genetic algorithm with an SDM auto-negotiation model constructed based on fuzzy constraints, thereby enhancing the effectiveness of SDM by addressing the problems posed by incomplete information environments and rapidly generating treatment plans with high mutual satisfaction.

**Results:**

A variety of negotiation scenarios are simulated in experiments and the proposed model is compared with other excellent negotiation models. The results indicated that the proposed model better adapts to multivariate scenarios and maintains higher mutual satisfaction.

**Discussion:**

The agent negotiation framework supports SDM participants in accessing treatment plans that fit individual preferences, thereby increasing treatment satisfaction. Adding GA opponent preference prediction to the SDM negotiation framework can effectively improve negotiation performance in incomplete information environments.

## 1. Introduction

Shared decision-making (*SDM*) is a treatment decision-making model proposed for humanitarian considerations and the needs of medical ethics (Cathy et al., 1997; Drake and Deegan, 2009; Stiggelbout et al., 2015), where at least one doctor and one patient participate in the process of making a treatment plan, which is based on information sharing. The resulting treatment plan considers the wishes of both parties. Unlike traditional decision-making models, such as the paternalistic model, the informed decision-making model, and the professional-as-agent model, SDM does not give decision-making power to either party but fairly combines the treatment preferences of doctors and patients (Cathy et al., 1997). To some extent, SDM improves the compliance and satisfaction of patients in the treatment process, which increases the effect of treatment (Pieterse et al., 2019; Fiorillo et al., 2020).

The concept of SDM was first proposed by Veatch in 1972 (Veatch, 1972). In 1997, Cathy (Cathy et al., 1997) further clarified its definition and characteristics. After more than 40 years of development, SDM has gradually formed a complete system in the West (Coulter et al., 2011, 2022), including a complete theoretical system (Makoul and Clayman, 2006), patient decision-making aids (Thomson et al., 2007; Elwyn et al., 2013), SDM evaluation tools applied to different scenarios (O'Connor, 1995; Simon et al., 2006; Scholl et al., 2012), and relevant legal and policy support. However, at present, SDM is still a new concept in many countries and regions (Huang et al., 2015), and the clinical practice of SDM is not as effective as expected. Many factors influencing the effectiveness of SDM have been explored. According to Bomhof-Roordink et al.'s (2019) study, which analyzed SDM models presented before September 2019, the exchange of information on treatment plans is key to SDM and also forms the basis for patient participation. Many studies have come to similar conclusions and noted that the medical literacy of the patient has a significant impact on the exchange of information (Shen et al., 2019; Loftus et al., 2020; Alsulamy et al., 2021). Doctors also play a crucial role in the exchange of information. Song and Wu (Song and Wu, 2022) suggest that doctors have a responsibility to elicit preferences from patients, which requires good communication skills. However, a factor that should not be ignored is the need for long-term doctor–patient communication (Beach and Sugarman, 2019; Caverly and Hayward, 2020). However, it is difficult for busy clinicians to find enough time to implement SDM during consultations. Therefore, the current obstacles in SDM practice can be placed into two categories: (1) doctors are under heavy time pressure, which leads to insufficient time for communication between doctors and patients, and (2) subjective factors of both doctors and patients significantly influence decision-making (Covvey et al., 2019; Shinkunas et al., 2020).

To solve the above problems, this study suggests integrating artificial intelligence (*AI*) into SDM to reduce unnecessary subjectivity in decision-making and the high time cost associated with manual negotiation. This method has been applied in the research of clinical decision support systems (*CDSSs*) (Osheroff et al., 2004; Magrabi et al., 2019; Yang et al., 2019), which are software that matches the patient's characteristics with existing medical knowledge so as to provide doctors with evaluation suggestions for patients. Such medical knowledge can be obtained from a computerized medical knowledge base or from historical diagnosis data mining using AI. Many studies (Bright et al., 2012; Sutton et al., 2020) have proven that the application of a CDSS can help doctors improve the efficiency of diagnosis and reduce medication errors, thereby reducing department costs and improving service quality. However, in the process of giving advice, a CDSS considers the patient's physiological characteristics instead of the patient's personal preferences. Loftus et al. (2020) suggest that most CDSS methods are black-box models and are in conflict with the concept of patient-centered care. Because patient preferences are not considered in the recommendations, it is possible that the predicted outcomes may differ significantly from the patient's preferences. Other studies (Deegan, 2010; Almario et al., 2018) have proposed computerized decision aids to narrow the knowledge gap between doctors and patients, which would help patients make more informed choices according to their preferences, but the abovementioned time pressure remains unresolved.

To better focus on doctor and patient preferences and reduce time pressures, this study constructed an intelligent negotiation framework to support decision-making based on the SDM model (Elwyn et al., 2012). First, in the model, the doctor informs the patient that reasonable options are available. Second, the doctor lists the options and clearly describes their potential harm and benefits. Finally, the doctor elicits an expression of preference from the patient and judges whether to make a decision or postpone it. Obviously, decision-related choices are directly related to patient preferences. Doctors give professional advice to support patients in decision-making, which reflects a preference derived from medical expertise and diagnostic experience. Therefore, such choices can be considered a problem of negotiation. In order to represent these two individual preferences, an “agent” is used to represent doctors and patients in negotiations, which is the key to automating the negotiation framework.

An agent refers to a computer system in a complex and changeable environment (Wooldridge and Jennings, 1995); it has autonomy and social ability and is able to respond to the environment. An agent is able to express knowledge, belief, intention, and goal-oriented behavior. In other words, it always attempts to retain maximum benefits for participants with the goal of promoting the success of negotiation. Agent-based auto-negotiation helps participants come to an agreement that can bring them as much benefit as possible with reduced time costs (Lomuscio et al., 2003). However, one important factor in a successful negotiation is that the agent adapts its own strategy to the available opponent's information. Participants in SDM have difficulty fully disclosing their preferences, which may be due to failure in building trust between the doctor and patient or the doctor's inadequate conversation skills to make the patient comfortable enough to express a preference. Hence, adding a component of opponent preference predictions to the negotiation framework is necessary.

Agent-based auto-negotiation has been applied in fields such as electronic trade, power trading, resource distribution, and supply-chain planning in recent years. Many negotiation models have been proposed for different domains, mainly focusing on offer evaluation, concession strategies, and opponent models.

Offer evaluations are quite different between linear and non-linear negotiation domains. For continuous and linear negotiation domains, a suitable linear function is usually designed to evaluate the offer, as seen in the study by Amini et al. (2020). However, dealing with the non-linear and discrete negotiation domain is more complicated. Yang and Luo (2019) proposed a method to evaluate offers by ranking demand. Mansour et al. (2022) presented a hybrid negotiation method that adopts different offer-generation mechanisms to tackle both quantitative and qualitative issues. The preference-based method solves quantitative issues by calculating the reservation intervals of the agent, and the fuzzy similarity method solves qualitative issues by finding the most similar counteroffer to the last offer from the opponent. However, these methods do not consider uncertainty in participant preferences.

The concession strategy determines the agent's behavior toward giving up interests, including the opportunity and interval of the concession if conflicts exist between participants. Such methods can be divided into time-based strategies and behavior-based strategies (Faratin et al., 1998). Mirzayi et al. (2021) proposed an opponent-adaptive concession method that creates a concession neighborhood around the target utility of each round, and the radius of the neighborhood growth rate is determined by the negotiation time. Mansour (2020) presented an imitation offer ration tactic that considers both the current concession behavior of the agent and that offered by its opponent.

Many studies use predictions of opponent preferences to accelerate the convergence of incomplete information negotiation and adopt various learning algorithms to improve the accuracy of opponent models, such as Bayesian algorithms (Sim et al., 2008; Pooyandeh and Marceau, 2014; Yi et al., 2021), neural networks (Zafari and Nassiri-Mofakham, 2016), and reinforcement learning (Bagga et al., 2021a). Most of the research and applications of agent-based automatic negotiation models focus on linear values such as electronic market transactions and power transactions. For SDM, there are many non-linear problems. For example, the severity of medical side effects is discrete and difficult to express with a definite value, which means that the agreement of SDM has a large and discrete value space, making it more difficult to learn about the preferences of opponents.

Genetic algorithms (*GAs*) are also an effective method of promoting agreement in negotiation (Holland, 1975; Matos et al., 1998; Gao and Chen, 2010; de Jonge and Sierra, 2016). GAs use Darwin's “survival of the fittest” theory to simulate the evolution of natural populations in order to find the optimal solution. GAs are efficient in searching and are closer to the global optimum solution when faced with a large solution space (Lambora et al., 2019). Thus, many studies have used them for value-space searches. Bagga et al. (2021b) proposed a method using a GA to find Pareto frontiers in solving the problem of making Pareto optimal bids under uncertain opponent preferences from a multi-objective optimization stance. However, this study does not predict opponent preferences, as it is not an incomplete information negotiation environment. Ayachi et al. (2018) used GA in electronic trading to predict the reservation values and deadlines of their opponents and then adjust the agent's bid strategy based on the predicted opponent model. Choudhary and Bharadwaj (2019) developed a group recommendation system based on multi-agent negotiation, where a GA is used in the negotiation and recommendation-generation phases. First, the GA is employed to find the offer of maximum utility for each agent in the group and then to determine the ranking of the minimum distance from the preferences of all agents. Few studies have used GAs to predict opponent preferences in complex and large negotiation domains, such as multiple issues and non-linear domains. Particularly, in SDM negotiations, a treatment plan often contains multiple linear or non-linear issues.

In summary, to address time pressures while focusing on patient preferences in SDM, this study presents an agent-based negotiation framework using fuzzy constraints and a GA (*ANFGA*). The chromosome coding method, fitness function, and evolutionary method of the GA are redesigned for the prediction of an opponent model in a complex negotiation domain. The contributions of this study are as follows:

- We establish an agent model of doctors and patients by taking problems that need to be agreed upon in SDM as negotiation issues. The agent describes the preferences of the participants and uses fuzzy membership to represent the benefits of each value (Lin et al., 2022).
- We use GA to solve the problems caused by an incomplete information environment. The GA takes the bid as an individual in the population and updates the cognition of the opponent through population evolution.
- In experiments, we compare the prediction results of the GA with the real preference settings of opponents to verify the effectiveness of our model, and we compare the performance with other excellent agent models to prove that our model has better performance in SDM.

The rest of this article is structured as follows: In Section 2, we describe the definitions of problems and introduce our proposed model, ANFGA. In Section 3, we evaluate the negotiation presented and compare it with other state-of-the-art agents. In Section 4, we conclude the paper and discuss future research directions.

## 2. Method

### 2.1. Auto-negotiation framework for SDM

There are two types of agents in SDM, *DA*, and *PA*, which represent doctors and patients, respectively. This study simulates a bilateral negotiation scenario, which means that only one pair of *DA* and *PA* are involved in the negotiation. The inputs of the model are the preferences of the doctors and patients, which are represented by a fuzzy membership function, and the number of functions is determined by the number of issues. The output of the model is a treatment plan, which is composed of multiple issue values. More details are provided in the section below.

#### 2.1.1. Negotiation statement

Negotiation issues, which are denoted as *I* = {*I*_1_, *I*_2_, …, *I*_*i*_, …, *I*_*n*_} here, indicate treatment plan choices such as period, cost, and side effects. Each issue has a finite set of *k* possible values, $I_{i}=\left(v_{1}^{i},v_{2}^{i},\ldots ,v_{j}^{i},\ldots ,v_{k}^{i}\right)$, which denote the options of a choice. Selecting a value for each issue forms an offer, *O* = (*v*^1^, *v*^2^, …, *v*^*i*^, …, *v*^*n*^), which represents an available treatment plan. All possible bid sets are called solution spaces. The Stacked Alternating Offers Protocol (Aydogan et al., 2017) is adopted in the framework, which means that the offers are provided in turn in the negotiation process by *DA* and *PA* until the negotiation concludes.

When receiving an offer from an opponent, the agent has to respond to the opponent with one of the following actions: *accept*, *offer*, or *reject*. The choice of action is based on the agent's preference and negotiation strategy, which are introduced in Section 3. Preference includes a set of weights, **ω** = {*w*_1_, *w*_2_, …, *w*_*i*_, …, *w*_*n*_}, as well as a set of satisfaction functions, **F** = {*F*_1_, *F*_2_, …, *F*_*i*_, …, *F*_*n*_}, where **ω** represents the level of importance that the participants attach to each issue, and **F** maps the participant's preference for each value in the issue to a real value. Thus, an aggregated satisfaction function that expresses participants' satisfaction with offer *O* is defined as follows:


(1) $$\begin{array}{l} \text{Ψ}\left(O\right)=\overset{n}{\underset{i=1}{\sum }}\;w_{i}^{*}F_{i}\left(v^{i}\;\right), \end{array}$$

where *w*_*i*_∈[0, 1] indicates the weight of the *ith* issue; $\overset{1}{\underset{n}{\sum }}w_{i}=1$; *F*_*i*_ is the satisfaction function of the *ith* issue, and *n* is the number of issues.

#### 2.1.2. Fuzzy constraint satisfaction

Most problems in SDM are difficult to describe with precise information. For example, the treatment period considered appropriate by the patient is often an interval rather than an exact value. Patients' expectations are also not evenly distributed over the interval values. In addition, there are many constraining relationships between different issues that are not precisely available but have a strong influence on the negotiation results. To describe and deal with such situations, a fuzzy theory proposed by Zadeh (1996) is integrated into the negotiation framework of SDM (Liu et al., 2020, 2022). Hence, the problem in the negotiation framework is formulated as solving a fuzzy constraint satisfaction problem (*FCSP*). Many studies (Safaeian et al., 2019; Bhuyan et al., 2021; Deng et al., 2021) have demonstrated that fuzzy constraints can be a good representation of the unclear and uncertain preference relationships of different decision-makers for common issues.

**Figure 1:**
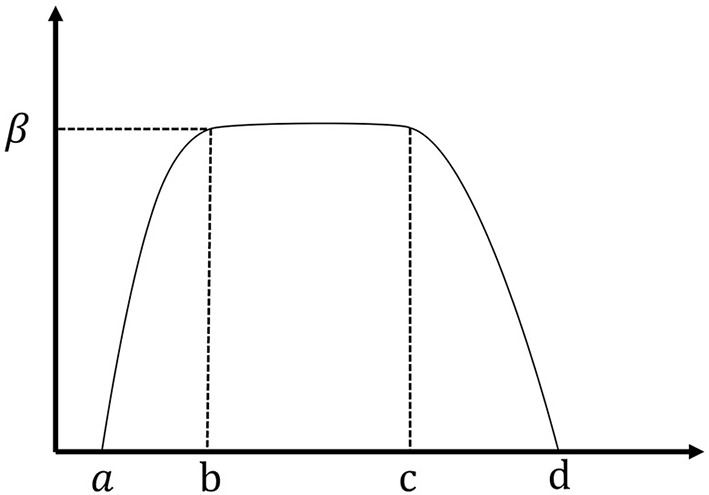
Trapezoid membership function image.

In our framework, participants' preferences are represented as a fuzzy membership function, *A*(*X*), which indicates the level of belonging of *X* to fuzzy set *A*. For preference in SDM, *X* is a solution option for the problem, and *A*(*X*)∈[0, 1] denotes the satisfaction with this option. In this way, the participants' uncertain preference for the problem is transformed into an accurate value. The trapezoid membership function is used in this study, as shown in Eq. (2) and Figure 1.


(2) $$A(X)=\mu_{i}\left(x\right)=\{\begin{array}{c} \;0,\;if\;x\;\leq \;a\\ \beta \left(1\;-\;{\left(\frac{x-b}{b-a}\right)}^{2}\right),\;if\;a<x<b\\ \;\frac{1}{\beta },\;if\;b\leq x\leq c\\ \beta \left(1\;-\;{\left(\frac{x-c}{c-d}\right)}^{2}\right),\;if\;c<x<d\\ \;0,\;if\;x\;\geq \;d \end{array}$$

Among them, the parameters *a, b, c, d*, and β determine the specific form of the function. *X*∈[*b, c*] can be expressed as the offer that the agent is most willing to accept. *X*<*a* and *X*>*d* represent the bid that the agent is most reluctant to accept. *X*∈[*a, b*] or *X*∈[*c, d*] represent different levels of satisfaction. Thus, Equation (1) can also be expressed as Equation (3):


(3) $$\begin{array}{l} \text{Ψ}\left(O\right)=\overset{n}{\underset{i=1}{\sum }}\;w_{i}^{*}\mu_{i}\left(v^{i}\right). \end{array}$$

### 2.2. Negotiation strategy

**Figure 2:**
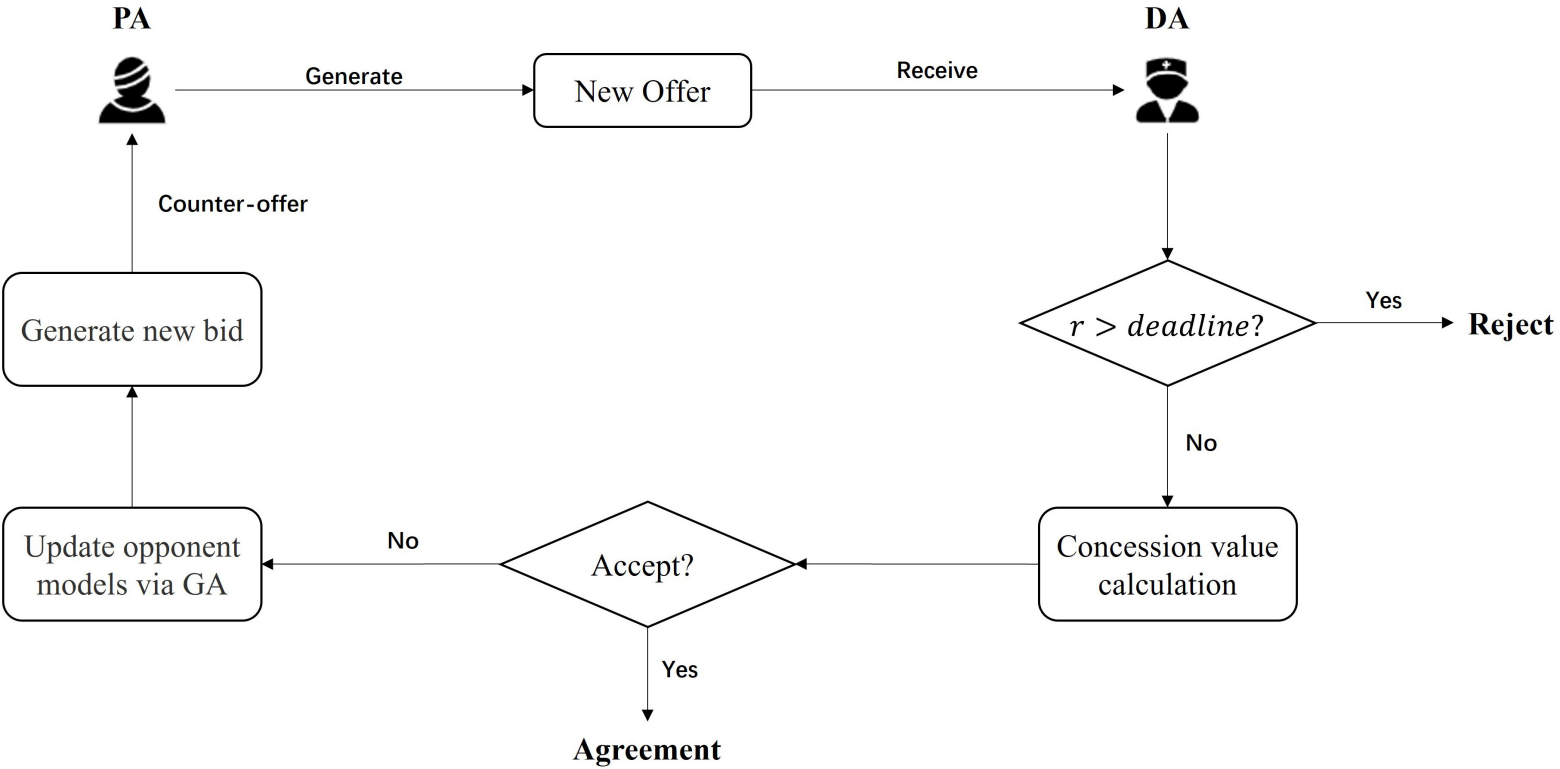
The process of ANFGA with a *PA* first offer.

Although the agent prefers a high-satisfaction offer, the opponents' preferences are frequently different or even contradictory. Appropriate concessions are necessary to prevent the failure of the negotiation. The negotiation strategy is designed to help the agent determine the appropriate concession pace and time, thereby increasing the success rate of negotiation and obtaining the maximum expected benefit. In incomplete information negotiation, the ability of opponent models to predict more information about opponents' preferences is an important factor in improving the efficiency of this process. Hence, an opponent model based on a GA is added to our model. The bidding process is similar between *DA* and *PA*. Figure 2 shows the progress of ANFGA with *PA* first offering a solution. *PA* sends an offer first, and if the current round is still before the deadline, the receiver calculates the concession value and decides whether to accept the offer. If accepted, the negotiation reaches an agreement, and the offer becomes the final solution. Otherwise, the counteroffer is generated and sent to the opponent via the GA update of the opponent model. In this section, we further introduce the negotiation strategist used in ANFGA, including the concession strategy and the opponent model.

#### 2.2.1. Concession strategy

Concession strategies help users reduce their expectations at the appropriate moment to promote successful negotiation. To make more sensible decisions, the agent calculates the pace of concessions by evaluating three states: the opponent's response state, the agent's own internal state, and the environment state. These states represent the opponent's desire, the agent's own desire, and the environmental constraints. The method used in this study improves on the work of Chia-Yu et al. (2016).

For the opponent's response state, *R*, the agent considers the difference between offer *A* generated in the previous round and the current offer *B* received from the opponent, as well as the initial offer, *A*_0_, and counteroffer, *B*_0_. The calculation is shown in Equation (4):


(4) $$\begin{array}{l} \sigma =1-\frac{G\left(A_{0},B_{0}\right)-G\left(A,\;B\right)}{G\left(A_{0},B_{0}\;\right)}. \end{array}$$

e*G*(*A, B*) is a measure of the distance between *A* and *B* for negotiation issue *I*_*i*_ϵ*X*, as shown in Equation 5:


(5) $$G\left(A,\;B\right)=\frac{\sqrt{\sum_{i=1}^{N_{i}}L{\left(A_{i},B_{i}\right)}^{2}}}{n},$$

where *A*_*i*_ and *B*_*i*_ denote the possibility distribution of *A and B* for negotiation issue *I*_*i*_ϵ*X*, and *n* denotes the number of negotiation issues.

The agent's own internal state, *M*, considers the level of satisfaction, ρ, associated with the latest offer, *A*, and its tightness with the acceptance threshold, ε, where


(6) $$\begin{array}{l} \rho =\text{Ψ}\left(A\right), \end{array}$$

(7) $$\begin{array}{l} \delta =1-\left(\rho -\;\varepsilon \right). \end{array}$$

The environmental constraint *E* to which the agent is subjected during the SDM negotiation process is primarily a time constraint. Therefore, it can be expressed as a function of time (Faratin et al., 1998), as shown in Equation 8:


(8) $$\begin{array}{l} \tau ={\lambda +\left(1-\lambda \;\right)\left(\frac{r}{r_{\max}}\right)}^{\frac{1}{\beta }}. \end{array}$$

In this equation, *r* is the current round, *r*_max_ denotes the negotiation deadline, and τ denotes the time constraint imposed on the agent during negotiation. λ∈[0, 1] represents the minimum concession value when first receiving a counteroffer from the opponent. If λ is large, the concession value will be high, and the acceptance threshold will be low at the beginning of the negotiation, which may lead to the agent easily accepting a low-satisfaction offer. β∈[0, 1] is the concession rate for time, controlling the pace at which the threshold falls. If β is low, the less the concession value decreases each round, and the slower the acceptance threshold decreases, which may result in more negotiation rounds.

Based on Equation (8), we can obtain the opponent response state, *R* = {σ}, the agent's internal state, *M* = {ρ, δ}, and the environment state, *E* = {τ}. Thus, we can calculate the concession value:


(9) $$\begin{array}{l} \varepsilon ={\left(\mu_{\rho }\left(\rho \right)\;\Lambda \mu_{\delta }\left(\delta \right)\;\Lambda \mu_{\sigma }\left(\sigma \right)\;\Lambda \mu_{\tau }\left(\tau \right)\;\right)}^{\omega }. \end{array}$$

The specific form of Equation (9) in this study is


(10) $$\begin{array}{l} \varepsilon ={\frac{\left(1-\frac{\sigma +\rho +\delta }{3}+\tau \right)}{4}}^{\omega }. \end{array}$$

Furthermore, the acceptance threshold of the agent at each round of negotiation can be calculated:


(11) $$\begin{array}{l} \varepsilon_{r}=\varepsilon_{r-1}-\varepsilon , \end{array}$$

(12) $$Action_{r}=\{\begin{array}{c} Accept,\;\Psi \left(O_{r}\right)\geq \varepsilon_{r}\\ Offer,\;\Psi \left(O_{r}\right)<\varepsilon_{r}\; \end{array}$$

where ε_*r*_ is the acceptance threshold of round *r*, *O*_*r*_ is the offer from the opponent in round *r*, and *Action*_*r*_ represents the response from the agent (Accept or Offer). If the satisfaction of *O*_*r*_ is more than ε_*r*_, the agent will accept the offer; otherwise, the agent generates and sends a new offer to the opponent.

From Eqs (9–11), it can be known that ω adjusts the rate of concessions. ω <1 implies a slower concession rate and expresses that the agent is unwilling to abandon too much interest in the negotiation, which represents a competitive concession strategy. ω = 1 implies a faster concessions rate and expresses that the agent wants to facilitate a quick agreement by reducing the benefits, which represents a collaborative concession strategy. ω>1 implies a win–win concession strategy, which lies between the first two strategies.

#### 2.2.2. Opponent model

**Figure 3:**
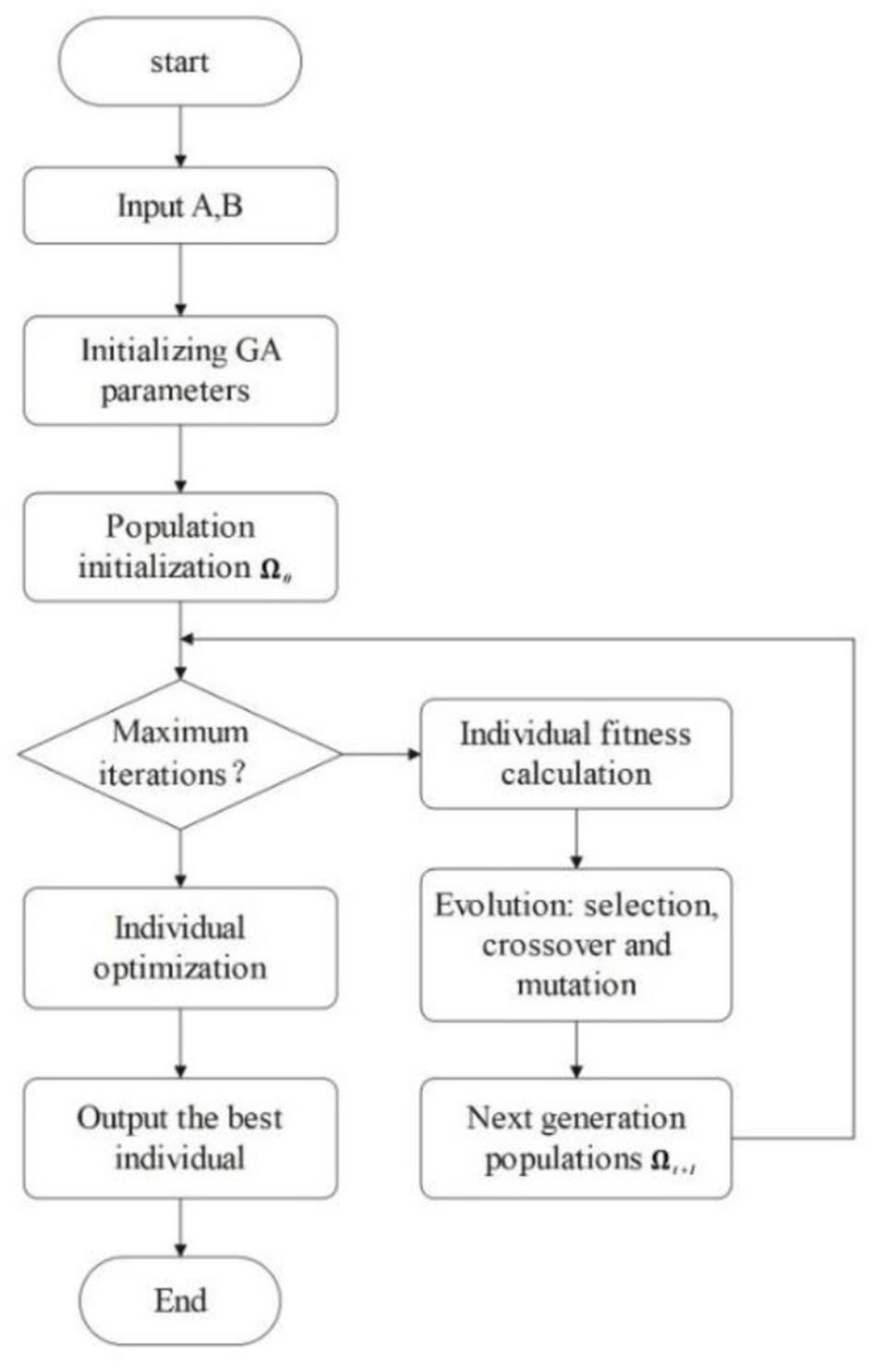
Opponent preference prediction process using GA.

As it is difficult for both sides of the SDM to publish their preferences accurately and clearly, negotiations are conducted in an incomplete information environment. Furthermore, opponent information is necessary to accelerate the rate of negotiation convergence. Therefore, we use a GA-based approach to learn information about an opponent's preferences. There are three important components of GA: population, fitness, and evolution. These will be described in detail in this subsection. Figure 3 shows the process of the method, where *A* and *B* are the histories of the agent and opponent offers.

##### 2.2.2.1. Population initialization

As defined in Section 2, the preference profile of the opponent has two parts: a set of weight preferences, **ω**, and a set of satisfaction functions, **F**. These parts form a binary set, Ω = {(*w*_1_, *F*_1_), ..., (*w*_*n*_, *F*_*n*_)}and *w*_*i*_∈**ω, ***F*_*i*_∈**F,** *i* = 1, ..., *n*, which represents the agent's preferences on each issue. Ω is a member of potential solution space, with multiple Ω forming a population **Ω = {**Ω_1_, Ω_2_, …, Ω_*j*_, …, Ω_*m*_**}**, where *m* is less than the size of the solution space.

The GA randomly generates *m* individuals to form the initial population, which is its starting point for searching for the optimal solution. In the two parts of Ω, we assign each weight *w*_*i*_ by generating random numbers between 0 and 1 and then regularizing them to initialize the weight preferences of Ω. For the satisfaction functions, we assume that the shape of the opponent preference function is consistent with that of the agent. Hence, five parameters are used to describe the opponent's satisfaction function: *a, b, c, d*, and β. Considering the agent's own preference (*a* and *d* are the boundary values that the agent is willing to accept on the issue) and the convergence speed of the opponent's preference population, the parameters *a* and *d* can be fixed and kept consistent with those of the agent. Therefore, the satisfaction preference information of Ω can be initialized by assigning random numbers to *b* and *c*, and β is fixed to 1.

##### 2.2.2.2. Fitness

The fitness function supports population evolution by evaluating the suitability of the individual in the current environment and current state. Excellent individuals will have a greater probability of being “inherited” by the next generation, which helps the population approach the optimal solution.

As the goal of SDM negotiations is to reach an agreement with the highest possible satisfaction for both parties, the fitness function can be defined as


(13) $$\begin{array}{l} f\left(\Omega_{j}\right)=f\left(\Omega^{k},\;\Omega_{j}^{\bar{k}}\right)=\frac{1}{N_{r}}\left(\overset{N_{r}}{\underset{r=1}{\sum }}{\text{Ψ}}^{k}{\left(A_{r}\right)}^{*}{\text{Ψ}}_{j}^{\bar{k}}\left(B_{r}\right)\right), \end{array}$$

where Ω_*j*_ is an individual of the opponent's preference population, Ω^*k*^ is the agent's preference profile, $\Omega_{j}^{\bar{k}}$ is the opponent's preference profile, *A*_*r*_ and *B*_*r*_ are the offers of the agent and the opponent in round *r*, respectively, and *N*_*r*_ is the current negotiation round.

##### 2.2.2.3. Evolution and generation of next populations

According to the principle of survival of the fittest, individuals with weak fitness in the population will be eliminated, whereas individuals with strong adaptability will survive and reproduce. In the algorithm proposed in this study, a certain number of excellent individuals are retained in the population evolution. The selection of individuals in the population adopts a method that combines an elite retention strategy with roulette selection.

The elite retention strategy refers to retaining a certain number of the best individuals in the population (the number in this method is *e*), as needed before individual crossover, which is directly inherited by the offspring population. This strategy method can prevent the optimal solution of a generation from being destroyed by crossover and mutation operations during the evolution process, thereby effectively improving the convergence of the GA.

Roulette selection, also known as proportional selection, refers to the probability of each individual being selected is proportional to its fitness. Its specific operations are as follows:

- Let Ω_*k*_ be an individual in the population, and its probability of being selected is:

(14) $$P\left(\Omega_{k}\right)=\frac{f(\Omega_{k})}{\sum_{j=1}^{m}f(\Omega_{j})}.$$

- The individual is given a random number, $r\in \left[0,\;\overset{m}{\underset{j=1}{\sum }}f\left(\Omega_{j}\right)\right]$; if $\overset{k}{\underset{j=1}{\sum }}f\left(\Omega_{k}\right)>$r, Ω_*k*_ joins the next generation population; otherwise, the loop continues.
- Repeat step b *m*−*e* times.

In order to ensure the diversity of the population, two excellent individuals from the population are selected as “parents” to generate new individuals (i.e., “crossover”). At the same time, there may be some “potential stocks” in the population, that is, individuals with a fitness level that is low currently but may increase after a few generations. To keep the potential stocks from being eliminated, they randomly get the chance to crossover. The parent individuals produce a child according to the crossover rules:

- The child copies partial preference information from the parents separately to form a complete individual preference.
- The child takes the average of the parents' preference information to form its own preferences.

In addition, there is a mutation rate that allows the child's partial preferences to possibly be assigned random values. This randomness can further help the model jump out of a local optimal solution and better approach the global optimal solution.

##### 2.2.2.4. Termination

Based on the GA, populations evolve to obtain the best individuals for their environment. During this process, the population terminates when it reaches the maximum number of iterations, namely, when it runs out of environmental resources and can no longer evolve. At this point, the best individuals from the latest generation are selected as the optimal solution.

##### 2.2.2.5. Optimal individual optimization

To avoid the uncertainty caused by multiple factors, a classification learning method is used to optimize the weighting information of the learned opponents. The specific process is as follows:

- The set of issues is divided into *n* categories based on the number of issues *n*: *C* = (*C*_1_, *C*_2_, …, *C*_*i*_, …, *C*_*n*_), where *C*_*i*_ is a concession on the issue of the current counteroffer and $\underset{I_{i}\epsilon I}{\sum }C_{i}$ is the sum of the concession values after several negotiations.
- Variable *c*, assigned to each category *C*, is used to mark the concession value of each variable on issue *I*.
- Suppose that *D*_*i*_ is the value domain of issue *i*, and the opponent makes a minimum concession each round in order to obtain a higher satisfaction value. In the multi-issue negotiation process, a larger overall concession for the issue implies a smaller weight. Hence, the proportional relationship between the weights can be expressed as

(15) $$\begin{array}{l} w_{1}:w_{2}:\ldots ,w_{i}:\ldots :w_{n}=\frac{1}{\frac{c_{1}}{D_{1}}}:\frac{1}{\frac{c_{2}}{D_{2}}}:\ldots :\frac{1}{\frac{c_{i}}{D_{i}}}:\ldots :\frac{1}{\frac{c_{n}}{D_{n}}}. \end{array}$$

- Performing the standard transformation, $\underset{1\leq i\leq n}{\sum }w_{i}=1$, we give weight to issue *I*_*i*_:

(16) $$w_{i}=\frac{\frac{\frac{1}{(c_{i}}}{D_{i}})}{\sum_{i=1}^{n}\frac{\frac{1}{(c_{i}}}{D_{i}})}.$$

- The final predicted opponent weights are expressed as follows:

(17) $$\begin{array}{l} w_{i}\;=\;\frac{w_{i}^{g}\;+w_{i}^{c}}{2}. \end{array}$$

As the negotiation proceeds, the agent's estimate of the opponent' issue weights is continuously updated and approaches the true weights.

## 3. Experiment and result

To evaluate the proposed model, we simulate experiments using the ANFGA for multiple negotiations in different scenarios. The measures used to evaluate the model and the results of the experiments are presented in this section.

### 3.1. Evaluation metrics

The following three common metrics are used in this paper to evaluate the proposed model:

- Average Joint Satisfaction is the average joint satisfaction of the two parties who finally reached the negotiation, which reflects the fairness of the negotiation and is calculated as follows:

(18) $$AJS=\frac{\sum_{t=1}^{T_{suc}}\Psi \left(A_{t}\right)+\Psi \left(B_{t}\;\right)}{T_{suc}},$$

where *T*_*suc*_ is the number of successful negotiations.

- The average negotiation round represents the speed of successful negotiation and is calculated as follows:

(19) $$ANR=\frac{\sum_{t=1}^{T_{suc}}R_{t}}{T_{suc}},$$

where *R*_*t*_ is the rounds of the *t*−*th* successful negotiation.

- Negotiation Success Rate represents the ratio of the number of successful negotiations to the number of negotiations and is calculated as follows:

(20) $$\begin{array}{l} NSR=\frac{T_{suc}}{T_{all}}, \end{array}$$

where *T*_*all*_ is the number of negotiations.

### 3.2. Experimental design

Table 1 shows the agent used in the experiment. To verify the validity of the performance of the negotiation model and the prediction of the opponent model, two types of experiments were designed:

- ANFGA is compared with ANF-TIME, which uses a time-based negotiation strategy under different time constraints and size solution spaces using three concession strategy types: competition (ω = 1.2), collaboration (ω = 0.8), and win–win (ω = 1).
- The negotiation results are compared using ANFGA in two different environments: incomplete information and complete information. Complete information means that participant preferences, including weights and satisfaction functions, are public. In the incomplete information environment, an additional FCAN is added as a reference for the performance improvement of the opponent model.

**Table 1:** Description of the agent used in the experiment.

**Agent**	**Negotiation strategy**
ANFGA-Competition	ANFGA uses a competitive strategy
ANFGA-Collaboration	ANFGA uses a collaborative strategy
ANFGA-Win-Win	ANFGA uses a win–win strategy
FCAN (Lin et al., 2022)	It uses the same concession strategy as the ANFGA but does not consider opponent preference prediction
ANF-TIME	Time-based negotiation strategy (Faratin et al., 1998) with fuzzy constraints
Complete information	ANFGA in a complete information environment

Each agent performs 200 times in the different experiments (i.e., *T*_*all*_ = 200). The two parameters of the concession strategy used in Equation (8) are set to λ = 0.1 and β = 0.25, which means that we assume that all ANFGA participants are not overly concerned with time constraints. The parameters for the GA of ANFGA are as follows: *Population*<*uscore*>*Size* = 100, *Max*_*Iteration*_ = 50, *Mutation*_*Rate*_ = 0.5, *and Elite*<*uscore*>*Rate* = 0.1,

which are determined by execution efficiency and model effectiveness.

The preference data used in the experiment are from the same questionnaire given in the Department of Pediatrics at Xiamen Hospital of Traditional Chinese Medicine as in the study by Lin et al. (2022). For the purpose of simulating more decision-making scenarios, including some extreme situations (e.g., a large number of issues or heavy time pressure), we generated more simulation preference data based on real data. Table 2 shows an example of preference data with five issues, which we use as input to demonstrate the model process more specifically. As shown in Table 2, preference is composed of the satisfaction function and weight for each issue, and the issue domain is below the issue name. Participants' satisfaction with each issue's value is determined by a trapezoid membership function, which is expressed as a four-tuple (Section 2.1.2). For example, *PA*'s satisfaction on issue *Cost* is (2, 3.5, 4, 6)_*F*_, meaning that the acceptable range of treatment cost is 2,000 to 6,000 RMB, and they are most willing to accept a treatment costing 3,500–4,000 RMB. Furthermore, weight is represented by a decimal value from 0 to 1, reflecting the importance of participants on the issue. For this case, the risk degree of the treatment plan is the most important for *PA*, whereas *DA* pays more attention to side effects.

**Table 2:** Participants' preference input on five issues.

**Issue**	**Preference**			
	**PA**		**DA**	
Cost (0–8k RMB)	(2, 3.5, 4, 6)_*F*_	(0.2)_*W*_	(4, 5, 7, 8)_*F*_	(0.25)_*W*_
Effective (1–10 rank)	(8, 9, 10, 10)_*F*_	(0.1)_*W*_	(6, 7, 8, 9)_*F*_	(0.15)_*W*_
Side-effects (0–100%)	(0, 0.05, 0.1, 0.2)_*F*_	(0.2)_*W*_	(0.1, 0.15, 0.2, 0.25)_*F*_	(0.3)_*W*_
Risk (0–100%)	(0, 0.02, 0.05, 0.15)_*F*_	(0.3)_*W*_	(0.05, 0.1, 0.15, 0.2)_*F*_	(0.2)_*W*_
Convenience (1–10 rank)	(8, 9, 10, 10)_*F*_	(0.2)_*W*_	(6, 7, 8, 9)_*F*_	(0.1)_*W*_

*F* means the satisfaction function of the issue, and *W* means the weight of the issue.

**Figure 4:**
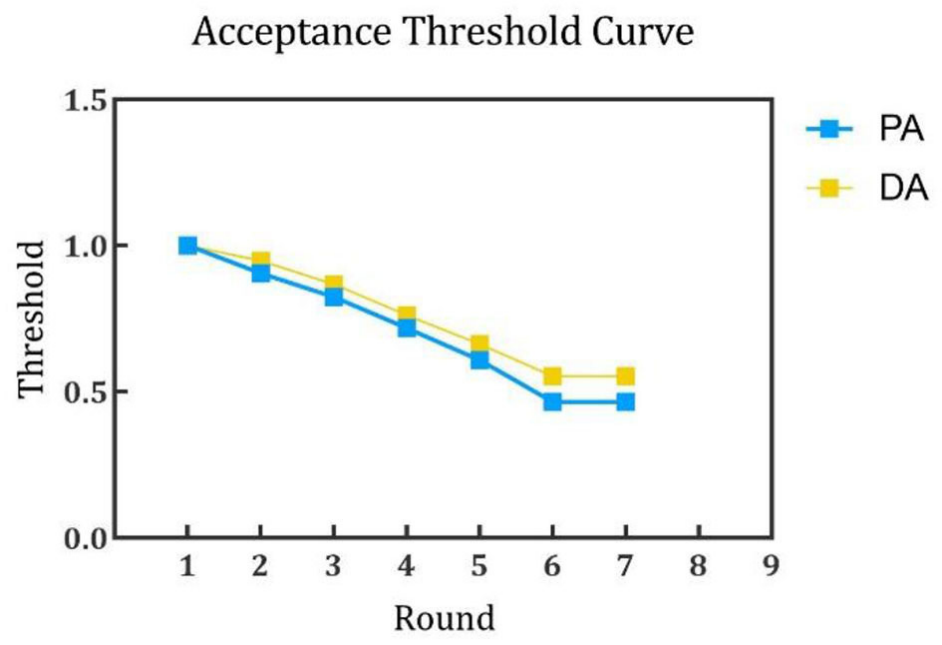
Acceptance threshold curve of *PA* and *DA*.

In addition, assuming that both *PA* and *DA* adopt collaboration strategies, the concession coefficient can be set as ω = 0.8, so that the concession value and acceptance threshold can be calculated by Eqs (4–11). Through the process described in Section 2, *PA* and *DA* reach an agreement after seven rounds of exchange offers, and the aggregate satisfaction of both participants is 0.76 and 0.75, respectively. The acceptance threshold curve in each round and more details of the agreement are shown in Figure 4 and Table 3, respectively. Thus, the doctor and patient can determine the specific treatment content by comparing the negotiated results with the actual disease treatment plan.

**Table 3:** Agreement.

**Issue**	**Agreement**
Cost	5.16
Effective	9
Side-effects	0.158
Risk	0.108
Convenience	9
Aggregate satisfaction	PA: 0.76, DA: 0.75

### 3.3. Experimental results

**Figure 5:**
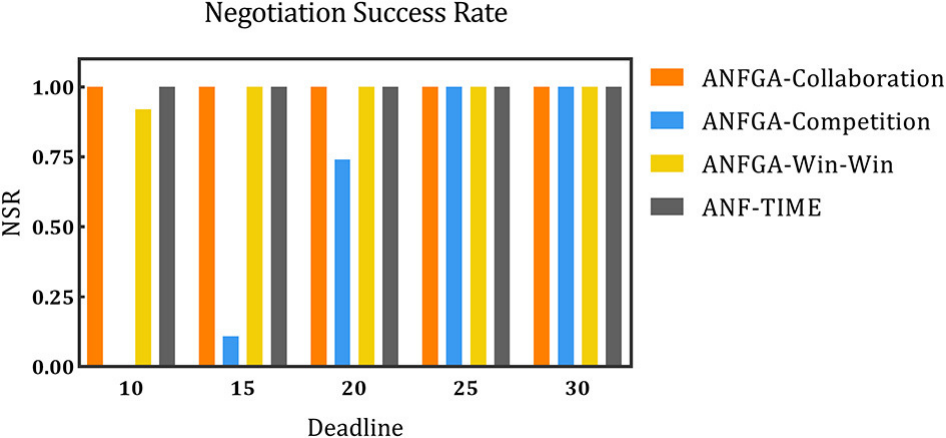
The negotiation success rate for the three concession strategy types for ANFGA and ANF-TIME in different deadlines.

**Figure 6:**
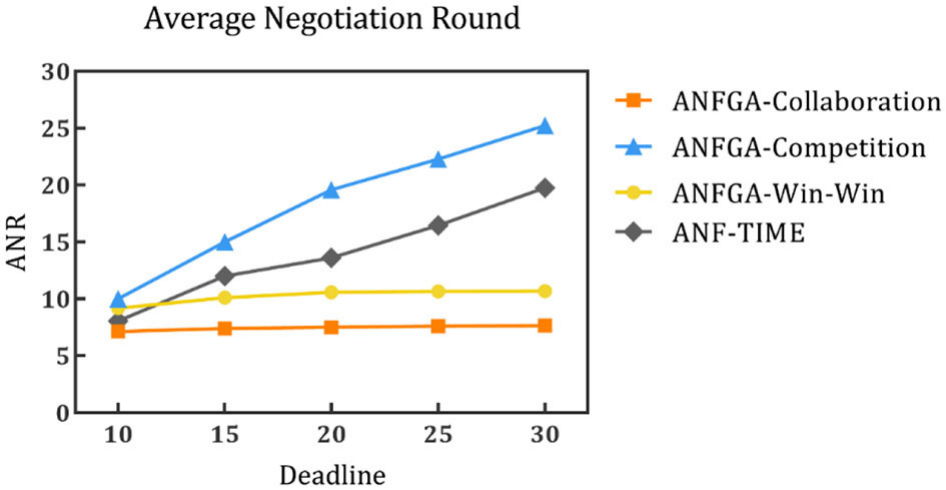
Average negotiation round for the three concession strategy types for ANFGA and ANF-TIME in different deadlines.

**Figure 7:**
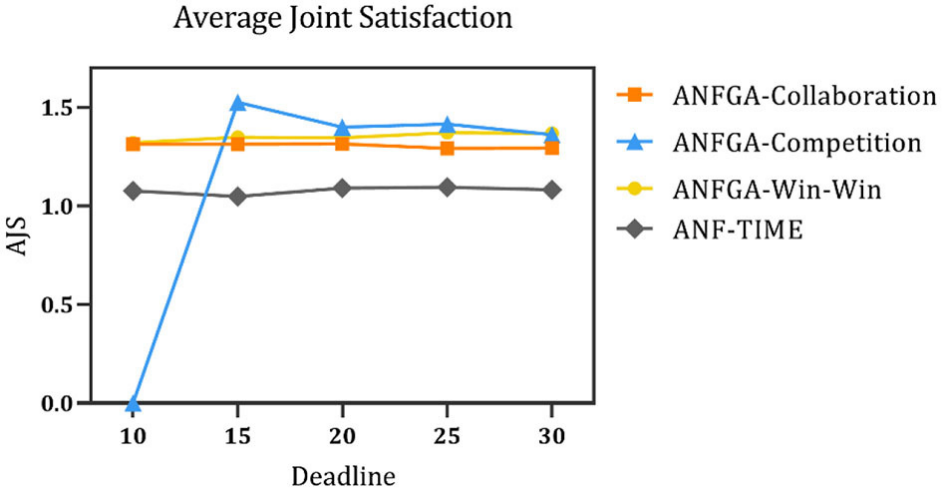
Average joint satisfaction for the three concession strategy types for ANFGA and ANF-TIME in different deadlines.

To simulate negotiation scenarios with different time constraints, the deadline for the number of negotiations is increased from 10 to 30, and the number of issues is fixed at *N* = 5. Figures 5–7 and Table 4 show the results for ANF-TIME and the three concession strategy types for ANFGA.

**Table 4:** AJS and ANR of ANF-TIME and three concession strategy types for ANFGA in different deadlines.

**Deadline**	**Metrics**	**ANF-TIME**	**ANFGA**		
			**Collaboration**	**Competition**	**Win–win**
10	AJS	1.076	1.313	0.000	**1.319**
	ANR	8.075	**7.130**	10.000	9.197
15	AJS	1.047	1.312	**1.525**	1.348
	ANR	12.010	**7.414**	15.000	10.110
20	AJS	1.089	1.313	**1.398**	1.346
	ANR	13.600	**7.500**	19.583	10.600
25	AJS	1.095	1.292	**1.415**	1.371
	ANR	16.460	**7.610**	22.295	10.665
30	AJS	1.082	1.293	1.361	**1.368**
	ANR	19.765	**7.665**	25.245	10.690

Bold means the best performance among all comparison items.

As Figure 5 shows, only ANFGA-Collaboration and ANF-TIME are able to maintain a 100% NSR at any deadline, and the other two agents show varying degrees of loss. In Figure 6, ANFGA-Collaboration requires the lowest ANR; in contrast, ANFGA needs more time to negotiate. Meanwhile, at larger deadlines, the ANR of ANF-TIME is more than that of ANFGA-Win-Win but still less than that of ANFGA-Competition. Figure 7 shows that ANFGA-Competition is able to reach the highest AJS under *deadline* = 15, whereas ANF-TIME maintains a lower AJS than the other two ANFGA types.

The above results show that ANFGA-Collaboration outperforms ANF-TIME for all metrics at any deadline, as ANF-TIME's concessions only depend on the deadline without considering retaining their own interests and updating strategies from the state of the environment or opponent. In addition, although ANFGA-Competition brings greater satisfaction to both parties of the negotiation, it also involves more time costs and a higher risk of failure, as competitive strategies make the agent willing to spend time to receive more benefit for itself.

**Figure 8:**
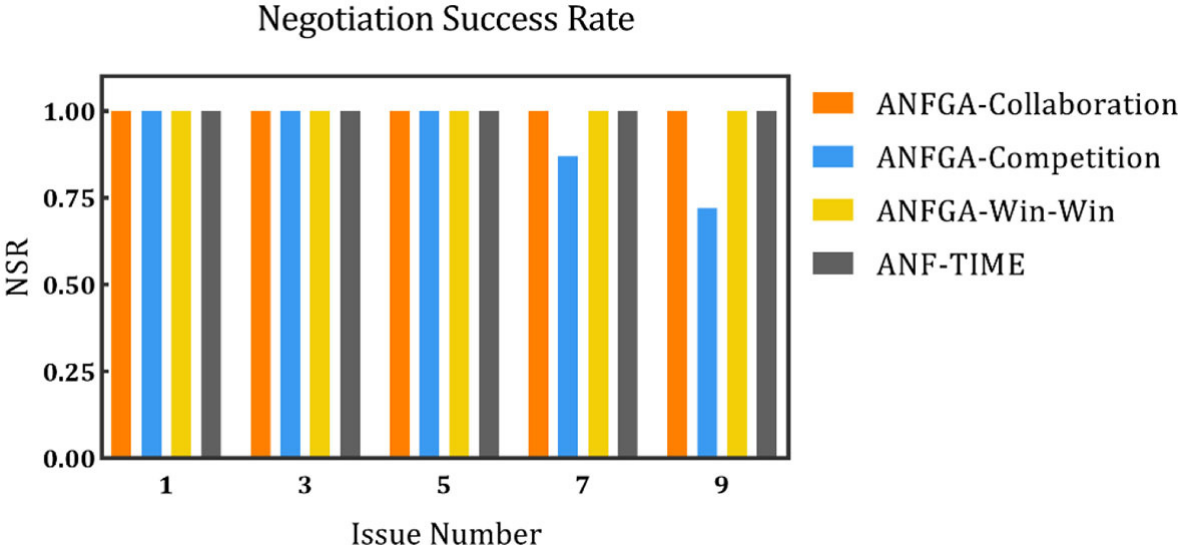
The negotiation success rate for the three concession strategy types for ANFGA and ANF-TIME in different issue numbers.

**Figure 9:**
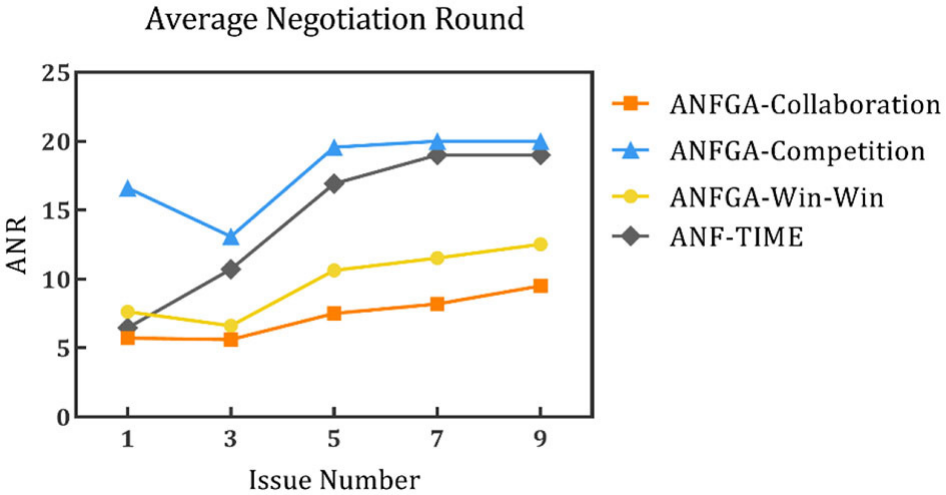
Average negotiation round for the three concession strategy types for ANFGA and ANF-TIME in different issue numbers.

**Figure 10:**
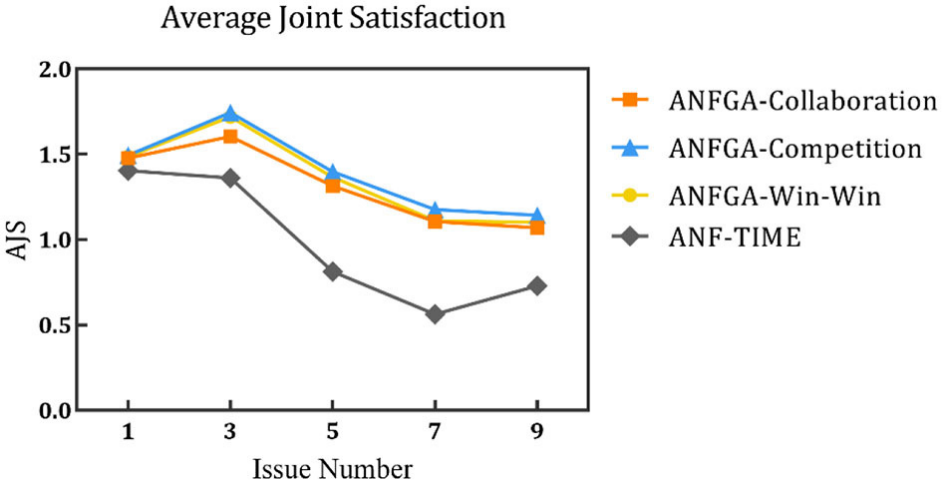
Average joint satisfaction for the three concession strategy types for ANFGA and ANF-TIME in different issue numbers.

To simulate the negotiation scenario in different solution spaces, the negotiation deadline was fixed at *N* = 20, and experiments were conducted with the number of issues being set at 1, 3, 5, 7, and 9. The results are shown in Figures 8–10 and Table 5.

**Table 5:** AJS and ANR of ANF-TIME and three concession strategy types for ANFGA in different issue numbers.

**Deadline**	**Metrics**	**ANF-TIME**	**ANFGA**		
			**Collaboration**	**Competition**	**Win–win**
1	AJS	1.405	1.477	**1.494**	1.486
	ANR	6.460	**5.720**	16.610	7.625
3	AJS	1.360	1.604	**1.744**	1.720
	ANR	10.715	**5.615**	13.085	6.605
5	AJS	0.812	1.313	**1.398**	1.365
	ANR	16.925	**7.500**	19.583	10.635
7	AJS	0.563	1.105	**1.177**	1.111
	ANR	19.000	**8.200**	20.000	11.527
9	AJS	0.729	1.069	**1.143**	1.101
	ANR	19.000	**9.500**	20.000	12.527

Bold means the best performance among all comparison items.

In Figure 8, all agents except ANFGA-Competition maintain 100% NSR under all issue numbers. In Figures 9, 10, similar to the first experiment, ANFGA-Competition is able to give the highest AJS agreement, but it still takes the most time. Furthermore, ANFGA-Collaboration outperforms ANF-TIME in all metrics.

From the above results, it can be seen that as the number of issues increases, the time required for negotiation becomes longer, and joint satisfaction decreases. Increasing the number of issues means the solution space becomes larger, making the search more difficult. At the same time, it takes more time for both parties to agree on all issues. Compared with ANF-TIME, ANFGA maintains a better and more stable performance in large solution spaces.

**Figure 11:**
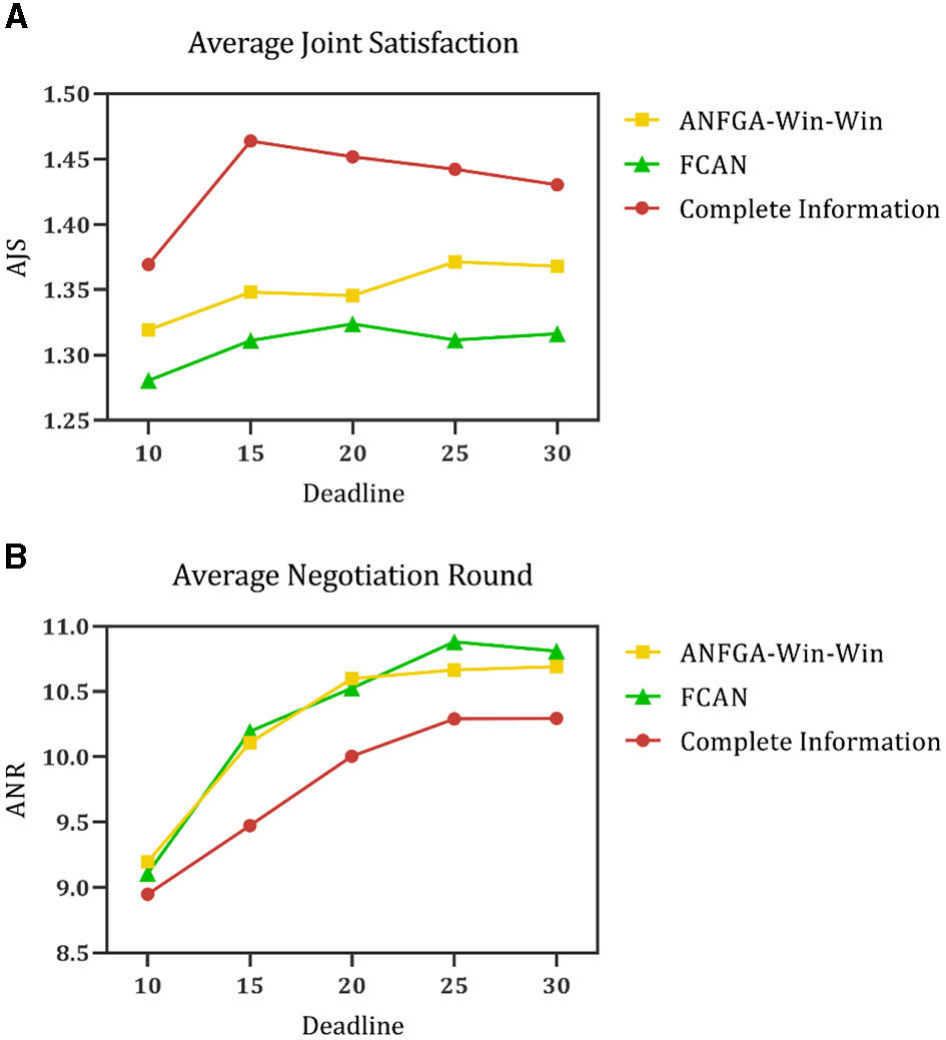
**(A)** Average joint satisfaction of negotiation between complete information and incomplete information in different deadlines. **(B)** Average negotiation round of negotiation between complete information and incomplete information in different deadlines.

**Figure 12:**
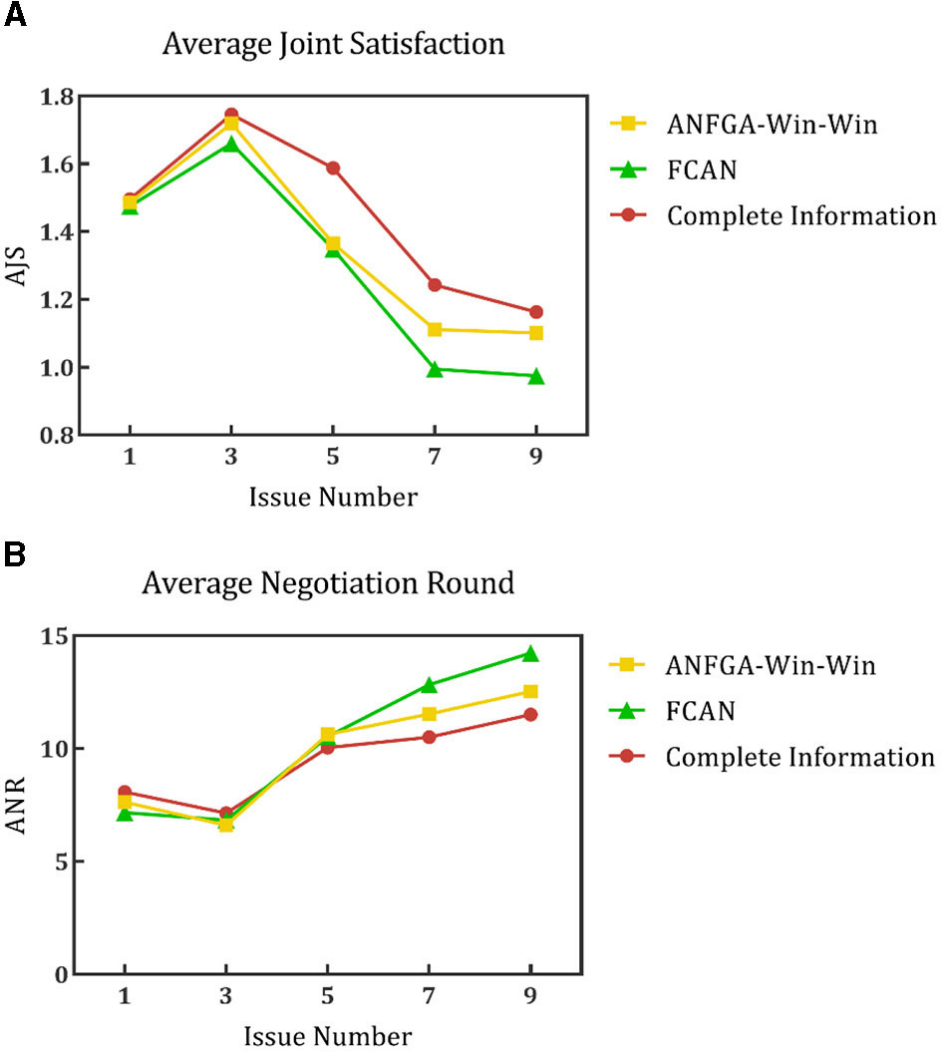
**(A)** Average joint satisfaction of negotiation between complete information and incomplete information in different issue numbers. **(B)** Average negotiation round of negotiation between complete information and incomplete information in different issue numbers.

To validate the performance of the proposed model for opponent preference prediction, we used the same strategy to negotiate in both complete and incomplete information environments. In the complete information environment, the agent is allowed to obtain the opponent's satisfaction function and weight of issues without prediction and then substitute them into Eqs (12), (16) directly. Figure 11 shows the results of this process at different deadlines. It can be seen that ANFGA has a better AJS compared with FCAN, but the former does not significantly improve ANR. ANFGA has ~0.1 distance to the complete information on both metrics. Figure 12 shows the negotiation results under different issue numbers. The distance between the three agents is smaller when the issue number is small, but when the issue number is large, ANFGA performs significantly better and is closer to the complete information than FCAN. As can be seen from the above results, the addition of the opponent model provides a more satisfactory agreement between the parties and decreases the number of rounds required for negotiation, which effectively mitigates the problems caused by an incomplete negotiation environment.

## 4. Discussion

Two experiments are conducted with three metrics to evaluate our model. The experimental results show that ANFGA has better performance than comparison work in both heavy time pressure and complex negotiation domains. Thus, our model is more adaptable to the real negotiation scenario of SDM. Among the three concession strategies of ANFGA, the results of the first experiment showed that the competition strategy required more negotiation rounds but possessed the highest joint satisfaction, the collaboration strategy needed the fewest rounds but had the lowest joint satisfaction, and the win–win strategy was a trade-off between the former two. These results are in accordance with our assumption that our negotiation strategy is effective in expressing the concession preferences of different participants. In practice, doctors and patients can adopt different concession strategy types depending on their expectations of the outcome. The results of the second experiment showed that the effect of ANFGA in the incomplete information environment is closer to the effect of the complete information environment than the comparison model, which demonstrates that our model can deal well with the problem of incomplete obtaining of opponent preferences in SDM.

## 5. Conclusion

SDM is considered an effective method for achieving patient-centered healthcare but is hampered by time constraints and personal subjective factors such as illiteracy of medical knowledge and lack of communication skills in its implementation. To reduce these negative effects and facilitate the implementation of SDM, this study proposes an agent-based auto-negotiation framework that aims for SDM participants to get close to the desired treatment plan with only a vague description of their preferences. For this purpose, to represent the uncertainty of doctor and patient preferences, a fuzzy member function is used to express this information. In addition, the above barriers also leave SDM in an incomplete information environment, with the preferences of the opponent being unavailable, making the SDM unsatisfactory and inefficient. Thus, GA-based opponent preference prediction was added to the negotiation framework, which helps the auto-negotiation model to converge faster and obtain a more satisfying solution. To verify the model, we performed simulated experiments with different information environments and different constraints. From the two metrics, AJS and ANR, it is evident that the proposed model has better performance than the agent without an opponent preference prediction strategy and remains stable under conditions of high time pressure and large solution space. The results also show that this model has promising potential when implementing the SDM between doctors and patients in real medical environments.

In the future, we will continue to maintain close contact with the clinic, collect relevant data, and conduct experiments in a real clinical environment in the next stage of our work. We also aim to improve the convergence and robustness of the model based on the results of its implementation.

## Data availability statement

The original contributions presented in the study are included in the article/supplementary material, further inquiries can be directed to the corresponding author.

## Ethics statement

The studies involving human participants were reviewed and approved by Medical Ethics Committee of Xiamen Hospital of Traditional Chinese Medicine. The patients/participants provided their written informed consent to participate in this study.

## Author contributions

K-BL and YL contributed to the conception, design, and first draft of writing. YW completed the code development and performed experiments. F-PH and Y-MY are responsible for data collection and interpretation. PL performed the statistical analysis. All authors contributed to the article revision and approved the final version.

## References

[B1] Almario C. V. Keller M. S. Chen M. Lasch K. Ursos L. Shklovskaya J.. (2018). Optimizing selection of biologics in inflammatory bowel disease: development of an online patient decision aid using conjoint analysis. Am. J. Gastroenterol. 113, 58–71. 10.1038/ajg.2017.470 29206816

[B2] Alsulamy N. Lee A. Thokala P. Alessa T. (2021). Views of stakeholders on factors influencing shared decision-making in the Eastern Mediterranean Region: a systematic review. East Mediterr. Health J. 27, 300–311. 10.26719/emhj.20.139 33788220

[B3] Amini M. Fathian M. Ghazanfari M. (2020). A BOA-based adaptive strategy with multi-party perspective for automated multilateral negotiations. Appl. Intell. 50, 2718–2748. 10.1007/s10489-020-01646-y

[B4] Ayachi R. Bouhani H. Amor B. (2018). An evolutionary approach for learning opponent's deadline and reserve points in multi-issue negotiation. Int. J. Interact. Multimed. Artif. Intell. 5, 131–140. 10.9781/ijimai.2018.08.001

[B5] Aydogan R. Festen D. Hindriks K. V. Jonker C. M. (2017). “Alternating offers protocols for multilateral negotiation,” in Modern Approaches to Agent-based Complex Automated Negotiation, eds K. Fujita, Q. Bai, T. Ito, M. Zhang, F. Ren, R. Aydogan, and R. Hadfi (New York, NY: Springer), 153–167. 10.1007/978-3-319-51563-2_10

[B6] Bagga P. Paoletti N. Alrayes B. Stathis K. (2021a). ANEGMA: an automated negotiation model for e-markets. Auton. Agent Multi. Agent Syst. 35, 1–28. 10.1007/s10458-021-09513-x19022721

[B7] Bagga P. Paoletti N. Stathis K. (2021b). “Pareto bid estimation for multi-issue bilateral negotiation under user preference uncertainty,” in 2021 IEEE International Conference on Fuzzy Systems (FUZZ-IEEE) (Luxembourg: IEEE), 1–6 10.1109/FUZZ45933.2021.9494429

[B8] Beach M. C. Sugarman J. (2019). Realizing shared decision-making in practice. JAMA 322, 811–812. 10.1001/jama.2019.9797 31343669 PMC8786261

[B9] Bhuyan H. K. Kamila N. K. Pani S. K. (2021). Individual privacy in data mining using fuzzy optimization. Eng. Optim. 54, 1305–1323. 10.1080/0305215X.2021.1922897

[B10] Bomhof-Roordink H. Gärtner F. R. Stiggelbout A. M. Pieterse A. H. (2019). Key components of shared decision-making models: a systematic review. BMJ Open 9, e031763. 10.1136/bmjopen-2019-031763 31852700 PMC6937101

[B11] Bright T. J. Wong A. Dhurjati R. Bristow E. Bastian L. Coeytaux R. R.. (2012). Effect of clinical decision-support systems: a systematic review. Ann. Intern. Med. 157, 29–43. 10.7326/0003-4819-157-1-201207030-00450 22751758

[B12] Cathy C. Amiram G. Tim W. (1997). Shared decision-making in the medical encounter: what does it mean? (or it takes at least two to tango). Soc. Sci. Med. 44, 681–692. 10.1016/S0277-9536(96)00221-3 9032835

[B13] Caverly T. J. Hayward R. A. (2020). Dealing with the lack of time for detailed shared decision-making in primary care: everyday shared decision-making. J. Gen. Intern. Med. 35, 3045–3049. 10.1007/s11606-020-06043-2 32779137 PMC7572954

[B14] Chia-Yu H. Bo-Ruei K. Van Lam H. Lai K. R. (2016). Agent-based fuzzy constraint-directed negotiation mechanism for distributed job shop scheduling. Eng. Appl. Artif. Intell. 53, 140–154. 10.1016/j.engappai.2016.04.005

[B15] Choudhary N. Bharadwaj K. K. (2019). Evolutionary learning approach to multi-agent negotiation for group recommender systems. Multimed. Tools Appl. 78, 16221–16243. 10.1007/s11042-018-6984-3

[B16] Coulter A. Collins A. Edwards A. Entwistle V. Finnikin S. Joseph-Williams N.. (2022). Implementing shared decision-making in UK: progress 2017-2022. Z. Evid. Fortbild. Qual. Gesundhwes. 171, 139–143. 10.1016/j.zefq.2022.04.024 35610131

[B17] Coulter A. Edwards A. Elwyn G. Thomson R. (2011). Implementing shared decision-making in the UK. Z. Evid. Fortbild. Qual. Gesundhwes. 105, 300–304. 10.1016/j.zefq.2011.04.014 21620325

[B18] Covvey J. R. Kamal K. M. Gorse E. E. Mehta Z. Dhumal T. Heidari E.. (2019). Barriers and facilitators to shared decision-making in oncology: a systematic review of the literature. Support. Care Cancer 27, 1613–1637. 10.1007/s00520-019-04675-7 30737578

[B19] de Jonge D. Sierra C. (2016). “GANGSTER: an automated negotiator applying genetic algorithms,” in Recent Advances in Agent-based Complex Automated Negotiation, eds N. Fukuta, T. Ito, M. Zhang, K. Fujita, and V. Robu (New York, NY: Springer), 225–234. 10.1007/978-3-319-30307-9_14

[B20] Deegan P. E. (2010). A web application to support recovery and shared decision-making in psychiatric medication clinics. Psychiatr. Rehabil. J. 34, 23. 10.2975/34.1.2010.23.28 20615841

[B21] Deng X. Lin Y. Zhuang H. (2021). Uncertain portfolio with Fuzzy investment proportion based on possibilistic theory. Eng. Lett. 29, 803–812.

[B22] Drake R. E. Deegan P. E. (2009). Shared decision-making is an ethical imperative. Psychiatr. Serv. 60, 1007–1007. 10.1176/ps.2009.60.8.1007 19648184

[B23] Elwyn G. Frosch D. Thomson R. Joseph-Williams N. Lloyd A. Kinnersley P.. (2012). Shared decision-making: a model for clinical practice. J. Gen. Intern. Med. 27, 1361–1367. 10.1007/s11606-012-2077-6 22618581 PMC3445676

[B24] Elwyn G. Lloyd A. Joseph-Williams N. Cording E. Thomson R. Durand M.-A.. (2013). Option grids: shared decision-making made easier. Patient Educ. Couns. 90, 207–212. 10.1016/j.pec.2012.06.036 22854227

[B25] Faratin P. Sierra C. Jennings N. R. (1998). Negotiation decision functions for autonomous agents. Rob. Auton. Syst. 24, 159–182. 10.1016/S0921-8890(98)00029-3

[B26] Fiorillo A. Barlati S. Bellomo A. Corrivetti G. Nicolo G. Sampogna G.. (2020). The role of shared decision-making in improving adherence to pharmacological treatments in patients with schizophrenia: a clinical review. Ann. Gen. Psychiatry 19, 43. 10.1186/s12991-020-00293-4 32774442 PMC7409631

[B27] Gao T. Chen P. (2010). “Research on the decision-making of multi-issue/attribute negotiation based on agent technology and the genetic algorithm,” in 2010 Chinese Control and Decision Conference (Xuzhou: IEEE), 3523–3528.

[B28] Holland J. H. (1975). Adaptation in *Natural* and *Artificial Systems. An Introductory Analysis with Applications to Biology, Control and Artificial Intelligence*. Ann Arbor, MI: University of Michigan Press.

[B29] Huang R. Gionfriddo M. R. Zhang L. Leppin A. L. Ting H. H. Montori V. M.. (2015). Shared decision-making in the People's Republic of China: current status and future directions. Patient Prefer. Adherence 9, 1129–1141. 10.2147/PPA.S82110 26273201 PMC4532212

[B30] Lambora A. Gupta K. Chopra K. (2019). “Genetic algorithm-A literature review,” in 2019 International Conference on Machine Learning, Big Data, Cloud and Parallel Computing (COMITCon) (Faridabad: IEEE), 380–384. 10.1109/COMITCon.2019.8862255

[B31] Lin K. Liu Y. Lu P. Yang Y. Fan H. Hong F.. (2022). Fuzzy constraint-based agent negotiation framework for doctor-patient shared decision-making. BMC Med. Inform. Decis. Mak. 22, 218. 10.1186/s12911-022-01963-x 35964129 PMC9375298

[B32] Liu W. Luo W. Lin X. Li M. Yang S. (2020). “Evolutionary approach to multiparty multiobjective optimization problems with common pareto optimal solutions,” in 2020 IEEE Congress on Evolutionary Computation (CEC) (Glasgow: IEEE), 1–9. 10.1109/CEC48606.2020.9185747

[B33] Liu Y. Lu P. Yang Y. Hong F. Lin K. (2022). “Modeling doctor-patient shared decision-making as fuzzy constraint-based agent negotiation,” in Proceedings of the 1st International Conference on Health Big Data and Intelligent Healthcare - ICHIH (Setúbal: SciTePress), 48–55. 10.5220/0011228100003438

[B34] Loftus T. J. Tighe P. J. Filiberto A. C. Efron P. A. Brakenridge S. C. Mohr A. M.. (2020). Artificial intelligence and surgical decision-making. JAMA Surg. 155, 148–158. 10.1001/jamasurg.2019.4917 31825465 PMC7286802

[B35] Lomuscio A. R. Wooldridge M. Jennings N. R. (2003). A classification scheme for negotiation in electronic commerce. Group Decis. Negot. 12, 31–56. 10.1023/A:1022232410606

[B36] Magrabi F. Ammenwerth E. McNair J. B. De Keizer N. F. Hyppönen H. Nykänen P.. (2019). Artificial intelligence in clinical decision support: challenges for evaluating AI and practical implications. Yearb. Med. Inform. 28, 128–134. 10.1055/s-0039-1677903 31022752 PMC6697499

[B37] Makoul G. Clayman M. L. (2006). An integrative model of shared decision-making in medical encounters. Patient Educ. Couns. 60, 301–312. 10.1016/j.pec.2005.06.010 16051459

[B38] Mansour K. (2020). A hybrid concession mechanism for negotiating software agents in competitive environments. Int. J. Artif. Intell. Tools 29, 2050016. 10.1142/S0218213020500165

[B39] Mansour K. Al-Lahham Y. Tawil S. Kowalczyk R. Al-Qerem A. (2022). An effective negotiation strategy for quantitative and qualitative issues in multi-agent systems. Electronics 11, 2754. 10.3390/electronics11172754

[B40] Matos N. Sierra C. Jennings N. R. (1998). “Determining successful negotiation strategies: an evolutionary approach,” in Proceedings International Conference on Multi Agent Systems (Cat. No. 98EX160) (Paris: IEEE), 182–189.

[B41] Mirzayi S. Taghiyareh F. Nassiri-Mofakham F. (2021). An opponent-adaptive strategy to increase utility and fairness in agents' negotiation. Appl. Intell. 52, 3587–3603. 10.1007/s10489-021-02638-2

[B42] O'Connor A. M. (1995). Validation of a decisional conflict scale. Med. Decis. Making 15, 25–30. 10.1177/0272989X9501500105 7898294

[B43] Osheroff J. A. Pifer E. A. Sittig D. F. Jenders R. A. Teich J. M. (2004). Clinical Decision Support Implementers' Workbook. Chicago, IL: HIMSS, 68.

[B44] Pieterse A. H. Stiggelbout A. M. Montori V. M. (2019). Shared decision-making and the importance of time. JAMA 322, 25–26. 10.1001/jama.2019.3785 31002337

[B45] Pooyandeh M. Marceau D. J. (2014). Incorporating Bayesian learning in agent-based simulation of stakeholders' negotiation. Comput. Environ. Urban Syst. 48, 73–85. 10.1016/j.compenvurbsys.2014.07.003

[B46] Safaeian M. Fathollahi-Fard A. M. Tian G. Li Z. Ke H. (2019). A multiobjective supplier selection and order allocation through incremental discount in a fuzzy environment. J. Intell. Fuzzy Syst. 37, 1435–1455. 10.3233/JIFS-182843

[B47] Scholl I. Kriston L. Dirmaier J. Buchholz A. Härter M. (2012). Development and psychometric properties of the shared decision-making questionnaire–physician version (SDM-Q-Doc). Patient Educ. Couns. 88, 284–290. 10.1016/j.pec.2012.03.005 22480628

[B48] Shen H.-N. Lin C.-C. Hoffmann T. Tsai C.-Y. Hou W.-H. Kuo K. N.. (2019). The relationship between health literacy and perceived shared decision-making in patients with breast cancer. Patient Educ. Couns. 102, 360–366. 10.1016/j.pec.2018.09.017 30270171

[B49] Shinkunas L. A. Klipowicz C. J. Carlisle E. M. (2020). Shared decision-making in surgery: a scoping review of patient and surgeon preferences. BMC Med. Inform. Decis. Mak. 20, 190. 10.1186/s12911-020-01211-0 32787950 PMC7424662

[B50] Sim K. M. Guo Y. Shi B. (2008). BLGAN: Bayesian learning and genetic algorithm for supporting negotiation with incomplete information. IEEE Trans. Syst. Man. Cybern. B Cybern. 39, 198–211. 10.1109/TSMCB.2008.2004501 19068440

[B51] Simon D. Schorr G. Wirtz M. Vodermaier A. Caspari C. Neuner B.. (2006). Development and first validation of the shared decision-making questionnaire (SDM-Q). Patient Educ. Couns. 63, 319–327. 10.1016/j.pec.2006.04.012 16872793

[B52] Song K. Wu D. (2022). Shared decision-making in the management of patients with inflammatory bowel disease. World J. Gastroenterol. 28, 3092–3100. 10.3748/wjg.v28.i26.3092 36051346 PMC9331519

[B53] Stiggelbout A. M. Pieterse A. H. De Haes J.C. (2015). Shared decision-making: concepts, evidence, and practice. Patient Educ. Couns. 98, 1172–1179. 10.1016/j.pec.2015.06.022 26215573

[B54] Sutton R. T. Pincock D. Baumgart D. C. Sadowski D. C. Fedorak R. N. Kroeker K. I.. (2020). An overview of clinical decision support systems: benefits, risks, and strategies for success. NPJ Digit. Med. 3, 1–10. 10.1038/s41746-020-0221-y 32047862 PMC7005290

[B55] Thomson R. G. Eccles M. P. Steen I. N. Greenaway J. Stobbart L. Murtagh M. J.. (2007). A patient decision aid to support shared decision-making on anti-thrombotic treatment of patients with atrial fibrillation: randomised controlled trial. BMJ Qual. Saf. 16, 216–223. 10.1136/qshc.2006.018481 17545350 PMC2464985

[B56] Veatch R. M. (1972). Models for ethical medicine in a revolutionary age. Hastings Cent. Rep. 2, 5–7. 10.2307/3560825 4679693

[B57] Wooldridge M. Jennings N. R. (1995). Intelligent agents: theory and practice. Knowl. Eng. Rev. 10, 115–152. 10.1017/S0269888900008122

[B58] Yang Q. Steinfeld A. Zimmerman J. (2019). “Unremarkable ai: fitting intelligent decision support into critical, clinical decision-making processes,” in Proceedings of the 2019 CHI Conference on Human Factors in Computing Systems (New York, NY: ACM), 1–11. 10.1145/3290605.3300468

[B59] Yang Y. Luo X. (2019). “A multi-demand negotiation model with fuzzy concession strategies,” in Artificial Intelligence and Soft Computing: 18th International Conference, ICAISC 2019, Zakopane, Poland, June 16–20, 2019. Proceedings, Part II 18 (Cham: Springer), 689–707. 10.1007/978-3-030-20915-5_61

[B60] Yi Z. Xin-gang Z. Yu-zhuo Z. (2021). Bargaining strategies in bilateral electricity trading based on fuzzy Bayesian learning. Int. J. Electr. Power Energy Syst. 129, 106856. 10.1016/j.ijepes.2021.106856

[B61] Zadeh L. A. (1996). “Fuzzy sets,” in Fuzzy sets, fuzzy logic, and fuzzy systems: selected papers by Lotfi A Zadeh. *World Sci*. 394–432. 10.1142/2895

[B62] Zafari F. Nassiri-Mofakham F. (2016). POPPONENT: highly accurate, individually and socially efficient opponent preference model in bilateral smulti issue negotiations. Artif. Intell. 237, 59–91. 10.1016/j.artint.2016.04.001

